# Fluid therapy in the perioperative setting—a clinical review

**DOI:** 10.1186/s40560-016-0154-3

**Published:** 2016-04-16

**Authors:** Anders Winther Voldby, Birgitte Brandstrup

**Affiliations:** Department of Surgery, Holbaek University Hospital, Smedelundsgade 60, 4300 Holbaek, Denmark

**Keywords:** Fluid therapy, Restricted, Goal-directed fluid therapy, Postoperative complications, Outcome of surgery, Third space, Third space loss

## Abstract

**Background:**

Perioperative hypovolemia and fluid overload have effects on both complications following surgery and on patient survival. Therefore, the administration of intravenous fluids before, during, and after surgery at the right time and in the right amounts is of great importance. This review aims to analyze the literature concerning perioperative fluid therapy in abdominal surgery and to provide evidence-based recommendations for clinical practice.

**Results:**

Preoperative oral or intravenous administration of carbohydrate containing fluids has been shown to improve postoperative well-being and muscular strength and to reduce insulin resistance. Hence, the intake of fluid (preferably containing carbohydrates) should be encouraged up to 2 h prior to surgery in order to avoid dehydration. Excessive intravenous fluid administration adds to tissue inflammation and edema formation, thereby compromising tissue healing.

During major abdominal surgery a “zero-balance” intraoperative fluid strategy aims at avoiding fluid overload (and comparable to the so-called restrictive approach) as well as goal-directed fluid therapy (GDT). Both proved to significantly reduce postoperative complications when compared to “standard fluid therapy”. Trials comparing “restrictive” or zero-balance and GDT have shown equal results, as long as fluid overload is avoided in the GDT group as well (categorized as “zero-balance GDT”).

It is possible that high-risk surgical patients, such as those undergoing acute surgery, may benefit from the continuous monitoring of circulatory status that the GDT provides. Data on this group of patients is not available at present, but trials are ongoing.

**Conclusion:**

In elective surgery, the zero-balance approach has shown to reduce postoperative complications and is easily applied for most patients. It is less expensive and simpler than the zero-balance GDT approach and therefore recommended in this review. In outpatient surgery, 1–2 L of balanced crystalloids reduces postoperative nausea and vomiting and improves well-being.

## Introduction

Intravenous fluid therapy is an integrated and lifesaving part of the treatment of patients undergoing surgery. Hypovolemia leads to insufficient circulation with decreased oxygen delivery to organs and peripheral tissues causing organ dysfunction and shock. Fluid overload, on the other hand, leads to interstitial edema and local inflammation and impairs the regeneration of collagen, thereby weakening the tissue healing with increased risk of postoperative wound infections, wound rupture, and anastomotic leakage. Moreover, it causes impaired cardiopulmonary function [1–14]. It is therefore imperative to administer fluid therapy individually, when needed, and in the right amounts [15, 16].

The goal of perioperative intravenous fluid therapy is to maintain or restore circulation with an adequate fluid and electrolyte balance, thereby creating the preconditions for a favorable outcome for the patient. Hence, the goals of perioperative fluid therapy can be summarized as follows:

- Maintain or correct fluid balance (dehydration, hypovolemia)
- Maintain or correct plasma constitution (electrolytes)
- Secure sufficient circulation (in combination with vasoactive and/or cardioactive substances)
- Secure sufficient oxygen delivery to organs (in combination with oxygen therapy)

In daily clinical practice, fluid therapy is guided by knowledge of basic physiological needs and simple cardiovascular measurements as well as the monitoring of the renal function by urine output. However, parameters such as mean arterial pressure (MAP), heart rate (HR), and diuresis are affected by variables not related to the circulatory status, including pain, body temperature, and physiological and psychological stress, as well as anesthetic and analgesic drugs, etc. These parameters are therefore imprecise in the measurement of intravascular status. The blood volume has to decrease by approximately 20 % before hypovolemia is detected, and fluid overload does not change blood pressure or HR at all in patients without heart failure. Therefore, using these parameters, fluid overload is invisible for the treating clinician giving intravenous fluid therapy to surgical patients, and its deleterious effects only become apparent in clinical trials avoiding fluid overload.

Consequently, the use of central cardiovascular measurements such as stroke volume or functional parameters (arterial wave form analysis, stroke volume variation, etc.) is recommended for the monitoring of circulatory status (goal-directed fluid therapy—GDT) to secure sufficient circulation and avoid fluid overload, with or without the simultaneous use of “zero-balance” or “restricted” fluid therapy.

The aim of this review is to analyze the literature concerning perioperative fluid therapy in abdominal surgery and to provide evidence-based recommendations for clinical practice.

## Review

### Fluid therapy preoperative

Fasting prior to surgery is mandatory to avoid aspiration of stomach content to the lungs. Six hours fasting from food and 2 h from liquids is generally recommended, and the patient should be encouraged to minimize the fasting period, thus avoiding dehydration [17].

Carbohydrates given orally or intravenously have been shown to improve postoperative well-being and muscular strength and to attenuate insulin resistance, the latter being correlated to prolonged length of hospital stay [18–20]. For this reason, this practice is indorsed, even when no effect on postoperative complications and mortality has been shown.

Jacob et al. show that a prolonged fasting period is unlikely to affect cardiopulmonary function and cause hypovolemia in healthy patients [21]. Thus, fasting deficit is not extensive for a patient who has been drinking up to 2 h prior to surgery. The loss is the combined fluid loss through diuresis and insensible perspiration and therefore primarily a loss of water which, if needed, should be compensated with glucose-containing fluids.

Mechanical bowel preparation prior to surgery has been argued to reduce postoperative leakage and infection. However, the benefit of the procedure has not been shown despite systematic review of the literature in a Cochrane review [22]. In addition, bowel preparation has been shown to induce functional hypovolemia affecting cardiovascular capacity and to cause preoperative dehydration [23]. Therefore, mechanical bowel preparation is no longer a standard recommendation.

### Fluid loss and replacement in the perioperative patient

Loss of fluid and electrolytes occurs continuously and has to be replaced to maintain homoeostasis. However, replacement regimes vary considerably within studies and unclear categorizations of perioperative fluid therapy as restrictive, conventional, or liberal creates confusion. In the earliest original papers testing the so-called restricted fluid therapy, the fluid regimen was in fact aiming at zero-balance measured as zero body-weight gain, thus, avoiding fluid overload. Therefore, the more descriptive term zero-balance is used in more recent papers from the same authors, as well as in this article. To ensure an optimal and adequate fluid replacement therapy, knowledge of physiological fluid turnover is fundamental.

#### Perspiration and diuresis

Several investigators have measured *insensible perspiration* (evaporation from the skin and the airways—the only loss of pure water from the body) in different circumstances. In 1977, Lamke et al. used a special chamber to measure the water content in the air layer immediately adjacent to the skin in four different zones of the body of adult healthy volunteers. They found insensible perspiration to be approximately 0.3 mL/kg/h [24]. Reithner et al. documented the same result for patients during abdominal surgery, but moreover showed that water loss from respiration was approximately 0.2 mL/kg/h. Thus, daily insensible perspiration amounts to approximately 0.5 mL/kg/h or 10 mL/kg/day [25–27]. During fever, insensible perspiration loss increases due to the rise of respiratory frequency. Reithner measured an increase in water loss from the respiratory tract of approximately 110 mL/day (0.06 mL/kg/h) in patients with fever above 39 °C [28]. However, taking into account that patients during surgery are ventilated with moist air, the insensible perspiration is only 0.3 mL/kg/h.

*Sensible perspiration* is visible sweat consisting of salt and water. The volume varies considerably depending on the surrounding temperature and physiological stress. Lamke et al. estimated visible sweat in patients with a rectal temperature above 39.5 °C to account for 600 mL/day (0.3 mL/kg/h). However, fever and sweating were occasional and only present for 6 h/day [29]. In a clinical setting, sensible perspiration is not generally considered, but may be significant for a patient with severe sepsis.

*Diuresis* is affected by a variety of factors including blood pressure, fluid intake, stress response (and other hormonal changes), surgical trauma, and anesthesia. Thus, diuresis reflects many other things than the renal ability to secrete fluid and osmotic components. Urinary output is therefore unreliable as a marker for intravascular fluid status and does not show the adequacy of the fluid therapy in the perioperative setting [30].

The expected diuresis for postsurgical patients varies in different countries, but a diuresis of 0.5–1.0 mL/kg/h is generally recommended. In several studies, the allowance of perioperative diuresis of 0.5 mL/kg/h in combination with a judicious fluid therapy has been shown to reduce postoperative morbidity [1, 5, 30].

When healthy individuals experience considerable thirst, the kidneys can concentrate urine to approximately 1200 mOsm/L and sodium in an amount of 300 mmol/L urine [31]. The clinical implication of this is illustrated in an average patient weighing 75 kg, not capable of drinking, and given 2 L 0.9 % saline as the only fluid therapy for a day. It is estimated that 750 mL of the water is lost as insensible perspiration, leaving 1250 mL to excrete 308 mmol sodium, hence bringing the kidneys close to their limit of sodium excretion. Age and diseases reduce the renal ability to concentrate diuresis, and infusion of large amounts of sodium is likely to cause unnecessary harm [32].

#### Intraoperative fluid losses and their replacement

Lamke et al. have measured the *evaporation from the surgical wound*. They used a chamber to cover the wound and the exteriorized viscera and found an evaporative loss correlating to the size of incision ranging from 2.1 g/h in minor wounds with slightly exposed viscera, up to 32 g/h in major wounds with completely exposed viscera [33]. An additional reduction by 87 % has been shown in a study on rabbits, using a plastic envelope covering the exposed viscera and irrigating the abdominal cavity with warmed crystalloids after replacement of the viscera to the abdominal cavity [34].

The evaporative fluid loss during laparoscopic surgery is considered small, yet dry air is insufflated into the abdomen with an unknown turnover. At present, evaporative loss during laparoscopic surgery is completely unknown.

#### The third space loss and the effects of intraoperative edema formation

It has been argued that surgical trauma leads to a shift of fluid volume between the fluid compartments of the body, creating a loss of extracellular fluid to a nonanatomical compartment named “the third space”.

This has led to the recommendation of giving up to 15 mL/kg/h the first hour of surgery and thereafter declining amounts of fluid in accordance with algorithms.

However, having reviewed the literature, this hypothesis is based on few studies using one specific but flawed method of measurement of the extracellular volume. More recent studies using sounder methods cannot demonstrate any such fluid loss. The entire concept of a loss to the third space should therefore be abandoned [35, 36].

Surgical trauma, however, does create an edema in the traumatized tissue as demonstrated by Chan et al. in 1983. They showed that the formation of a small bowel anastomosis in rabbits caused an increase in tissue weight of 5–10 %, due to fluid accumulation. Supplementary intravenous crystalloid infusion of 5 mL/kg/h doubled the edema and destabilized the anastomosis [37].

Transferring these findings to a clinical setting, a hypothetic manipulation of the entire colon (approximately 3 kg) results in water accumulation in the tissue of about 150–300 mL. Substituting this volume, additional edema formation appears, compromising the healing of anastomosis and increasing the risk of leakage [3, 10]. Moreover, the estimated maximal volume loss of 300 mL is very small and hardly causes a need for replacement [35].

Noblett et al. randomized 108 patients undergoing colorectal resection to intraoperative GDT compared to standard fluid therapy (3638 mL vs. 3834 mL) and showed that GDT significantly reduced interleukin 6 levels. This indicates that through securing splanchnic circulation by GDT, a reduction of the systemic inflammatory response due to surgical trauma was achieved [16]. In addition, in a study by Kulemann et al., excessive intraoperative intravenous administration of crystalloids was shown to promote inflammation and accelerated collagenolysis in rats [3]. These findings suggest that unrestrained administration of intravenous crystalloids induces adverse inflammatory responses and compromises wound healing.

The balance between sustaining intravascular volume and avoiding extravascular fluid accumulation is delicate. Lobo et al. infused 1 L saline and demonstrated that 68 % had escaped from the intravascular space 1 h after the infusion, compared to 16 % after the infusion of 1 L colloid [38]. Likewise, patients with moderate hypovolemia receiving rapid infusion of 1 L Ringers solution do not increase the intravascular volume compared to rapid infusion of 1 L hydroxyethyl starch 6 % (HES), which significantly improved blood expansion and cardiac output [39]. This suggests that crystalloids leave the intravascular volume fast and induce interstitial edema.

Acetated or lactated Ringers solutions are originally developed from the plasma of amphibians, but are closer to the composition of human plasma than saline. It contains less chloride than saline (100 mmol vs. 154 mmol) but still 140 mmol of sodium. Even though chloride causes hyperchloremic acidosis if given in excess amounts, the importance of sodium in the development of postoperative edema is unknown.

The use of colloids for stroke volume optimizing regimes has been shown to reduce postoperative complications [13, 40–42]. However, a recent study showed significant coagulopathy and adverse kidney effects using HES to stabilize patients with sepsis at intensive care units [43] and calls for caution using colloids for resuscitation. At the same time, a recent systematic review found no association between the use of starch solutions and acute kidney injury in surgical patients [44]. Therefore, the use of colloids in the perioperative setting seems safe.

Interstitial edema following intravenous fluid administration is formed and sustained as a result of osmotic forces and caused by the diffusion of osmotic active components, primarily excessive sodium and chloride infusion. This means that interstitial edema is not caused by excess of water (hydra = water) but “excess of salt” and should be treated as such. It is important to keep in mind that excess sodium is excreted slower than water [9, 32].

Oxygenation of organs is essential to preserve tissue function and avoid negative implications for wound healing and further complications. The correlation between oxygenation and sufficient circulation is subtle, since fluid optimization causes hemodilution and increases interstitial oedema, thereby compromising oxygen supply [45, 46]. Many methods have been tested to improve tissue oxygenation, but the invasive techniques limit clinical use [47, 48]. However, it is worth noting that in a randomized study by Jhanji et al., a significant increase in microcirculation and oxygenation of tissue was observed in patients receiving postoperative stroke volume-guided fluid therapy in combination with dopexamine. However, no difference in overall complications, a decrease in length of hospital stay (LOS) or inflammatory markers, was seen [49].

#### Liberal-, restricted-, or goal-directed approach

Belief in the existence of a third space loss and the fear of hypovolemia has led to a perioperative fluid practice of giving a large volume of intravenous fluid. However, observational studies show that a postoperative weight gain had deleterious side effects [2, 12, 50, 51] and formed the hypothesis behind the so-called restrictive fluid therapy, simply meaning avoiding fluid overload.

In a study of 141 patients undergoing colorectal surgery, Brandstrup et al. showed a beneficial effect of a more restrictive vs. a standard (liberal) fluid regimen (2740 mL vs. 5388 mL), reducing overall, major and minor postoperative complications and confirming that fluid overload caused poor tissue healing and cardiopulmonary complications [1]. This restrictive regimen aimed at zero-balance, measured as no more than 1 kg of body weight increase, and is also described as zero-balance fluid therapy. Similarly, Nisanevich et al. randomized 152 patients undergoing elective intraabdominal surgery to a restrictive vs. a standard (liberal) fluid regimen (1230 mL vs. 3670 mL), showing reduced complications, length of hospital stay, and faster bowel movement in the restrictive group [6]. Several subsequent trials have confirmed these results, all showing the benefits of a zero-balance perioperative fluid approach [2–5, 8, 9].

However, the fear of occult hypovolemia caused by a too restrictive fluid regimen [15] and the difficulty of handling the goal of zero-balance in unstable patients has led to the request for a hemodynamic goal. Different hemodynamic goals to direct the fluid therapy have been suggested, for example, arterial wave form analysis, central venous pressure, or lactate. In this review, GDT refers to studies using dynamic parameters such as stroke volume or pulse pressure variation analysis as goals during fluid optimization. The GDT approach has in several studies shown to improve outcome and reduce LOS and overall complications [16, 41, 42, 52–55]. The randomized trials of GDT in abdominal surgery are shown in Table 1 [14, 16, 41, 42, 52–59].

**Table 1:** Trials of “goal-directed fluid therapy” (GDT) in abdominal surgery versus “standard therapy”

Author	Surgery	No. of patients/ASA	Blinding/monitor/timing	Primary outcome	Intervention	Preoperative fluid volume, mL (control vs. GDT)	Intraoperative fluid volume, mL (control vs. GDT)	Postoperative fluid volume, mL (control vs. GDT)	Effect of GDT
Conway et al. [56]	Elective bowel surgery	57/ASA I-III	No blinding/ODM (CardioQ®)/intraoperative	Cardiac output	Optimizing SV (<10 %) and cFT (<350 ms) with HES 6 %	Not given	Coll: 19 mL/kg vs.28 mL/kg Total: 55 mL/kg vs. 64 mL/kg	Not given	↑ SV and CO → LOS → Complications ↓ Critical care admission Mortality: 1 (control) vs. 0 (GDT)
Gan et al. [55]	Elective major urological or gynecological	100/ASA I-III	No blinding/ODM (CardioQ®)/intraoperative	LOS	Optimizing cFT (<350 ms) and SV (<10 %) with HES 6 %	Not given	Coll: 282 vs. 847 Cryst: 4375 vs. 4405 Total^a^: 4775 vs. 5420	Not given	↓ LOS → Complications ↓ PONV Mortality not reported.
Wakeling et al. [42]. 2005	Elective colorectal resection	128/ASA II (media)	Observer blinded/ODM (CardioQ®)/intraoperative	LOS	Optimizing SV (<10 %) with Haemaccel® or Gelofusine®	1000–2000 Hartmann’s solution from midnight	Coll: 1500 vs. 2000 Cryst: 3000 vs. 3000 Total: not given	Not given Early oral intake	↓ LOS ↓ Complications ↓ GI complications Mortality: d30: non; d60: 1 (control) vs. 0 (GDT)
Noblett et al. [16]	Elective colorectal resection	108/ASA II (median)	Observer blinded/ODM (CardioQ®)/intraoperative	LOS	Optimizing SV (<10 %) and cFT (<350 ms) with Volplex®	Not given	Coll: 1209 vs. 1340 Cryst: 2625 vs. 2298 Total: not given	Not given Early oral intake	↓ LOS ↓ Complications ↓ Critical care admission ↓ Interleukin 6 response Mortality: 1 (control) vs. 0 (GDT)
Lopes et al. [52]	Elective mixed GI and urological	33/ASA II-IV	No blinding/IBPplus®/intraoperative	LOS	Optimizing PPV (≤10 %) with HES 6 %	Not given	Coll: 0 vs. 2247 Cryst: 1563 vs. 2176 Total^a^: 1694 vs. 4618	Not given Patients transferred to ICU	↓ LOS ↓ Complications ↓ Mechanical ventilation ↓ ICU stay Mortality: 5 (control) vs. 2 (GDT)
Buettner et al. [57]	Elective general, urological, or gynecological	80/ASA I-III	Not blinded. PiCCOplus®/intraoperative	ScvO_2_ and serum lactate	Optimizing SPV (<10 %) with HES 6 %, 130/0.4 (Voluven®) and vasopressors	Not given	Coll: 1000 vs. 1500 Cryst: 4250 vs. 4500 Total: not given	Not given	→ ScvO_2_ or lactate → Complications → Mechanical ventilation → ICU stay Mortality: 1 (control) vs. 0 (GDT)
Forget et al. [58]	Elective mixed GI surgery	82/ASA II-III	Observer blinded/Masimo Set®/intraoperative	Whole blood lactate levels	Optimizing PVI (>13 %) with HES 6 %, 130/0.4 (Voluven®) and vasopressors	Not given	Coll: 1003 vs. 890 Cryst: 1815 vs. 1363 Total^a^: 2918 vs. 2394	48 h postop. Coll: 358 vs. 268 Cryst: 3516 vs. 3107	↓ Lactate levels → Complications → Renal function Mortality: 0 (control) vs. 2 (GDT)
Mayer et al. [41]	Elective mixed GI surgery	60/ASA III	Observer blinded/FloTrac®, Vigileo/intraoperative	LOS	Optimizing CI (≥2.5 L/min/m^2^) with crystalloids, colloids, inotropes and vasopressors	Not given	Coll: 817 vs. 1188 Cryst: 3153 vs. 2489 Total^a^: 4494 vs. 4528	Not given	↓ LOS ↓ Complications → Mechanical ventilation → ICU stay Mortality: 2 (control) vs. 2 (GDT)
Benes et al. [54]	Elective mixed GI and vascular surgery	120/ASA II-IV	Observer blinded/FloTrac®, Vigileo/intraoperative	Complications	Optimizing SVV (<10 %) with HES 6 %, 130/0.4 (Voluven®) and inotropes	Not given	Coll: 1000 vs. 1425 Cryst: 2459 vs. 2321 Total^a^: 3729 vs. 3746	8 h postop: Coll: 0 vs. 0 Cryst: 1528 vs. 1587	↓ Complications → ICU stay → LOS ↓ Lactate levels Mortality: 2 (control) vs. 1 (GDT)
Challand et al. [59]	Elective open or laparoscopic colorectal surgery	179 subdivided into: fit (123) vs. unfit (56)/ASA I-IV	Observer blinded/ODM (CardioQ®)/intraoperative	LOS	Optimizing SV (<10 %) with HES 6 %, 130/0.4 (Voluven®)	971 vs. 1273 Hartmann’s solution	Coll: 336 vs. 1718 Cryst: 3593 vs. 3479 Total^a^: 4010 vs. 5309	1 postop. day: Fluid balance: 2011 vs. 2083	Unfit patients: → LOS → Complications Fit patients: ↑ LOS ↑ ICU admission → Complications Mortality: d30: 2 (control) vs. 2 (GDT); d90: 4 (control) vs. 5 (GDT)
Salzwedel et al. [53]	Elective general, urological, or gynecological	160/ASA II-III	Patient blinded/ProAQT®, PULSION®/intraoperative	Complications	Optimizing PPV (<10 %) and CI (≥2.5 L/min/m^2^) with fluids, vasopressors and inotropes	Not given	Coll: 725 vs. 774 Cryst: 2680 vs. 2862 Total: not given	24 h postop. Coll: 147 vs. 57 Cryst: 3452 vs. 3204	↓ Complications → LOS → Stay in postop. semi intensive care → Bowel movement Mortality: not given
Pearse et al. [14]	Planned/urgent GI surgery	734/ASA I-IV	No blinding/LiDCOrapid®/intraoperative and 6 h postop.	Complications and mortality d30	Optimizing SV (<10 %) with any colloid and dopexamine	Not given	Coll: 500 vs. 1250 Cryst: 2000 vs. 1000 Total^b^: 4024 vs. 4190	Coll: 0 vs. 500 Cryst: 600 vs. 506	→ Mortality and complications d30 → Complications d7 → Mortality d30 Mortality: d30: 11 (control) vs. 12 (GDT); d180: 42 (control) vs. 28 (GDT)

*ICU* intensive care unit, *PONV* postoperative nausea and vomiting, *LOS* length of hospital stay, *ODM* oesophageal Doppler monitoring, *CI* cardiac index, *SV* stroke volume, *SVV* stroke volume variation, *SPV* systolic pressure variation, *PPV* pulse pressure variation, *PVI* pleth variability index, *cFT* corrected flow time, *CVP* central venous pressure

^a^Total volume infused including colloid, crystalloid and blood products

^b^Total volume infused including colloid, crystalloid, blood products and intravenous medicine during intervention

↑significantly increased, ↓ significantly decreased, → no significant changes

A common factor to the trials on fluid therapy is that blinding is difficult since edema and diuresis is evident for all parts of treating patients. In addition, in all research concerning the surgical patient, many variables affect outcome and are difficult to standardize. Small sample sizes in the presented GDT trials challenge the results potentially affected by confounders. Furthermore, primary outcomes are dominated by LOS, which is a weak parameter influenced by local traditions and doctor and patient preferences and expectations.

Lopes et al. randomized 33 patients undergoing high-risk surgery to GDT vs. standard care (4618 mL vs. 1694 mL), perceiving the benefit of GDT with significant reduction in LOS, fewer patients developing complications, and shorter duration of mechanical ventilation [52]. In a study by Gan et al., patients receiving GDT were shown to reduce LOS compared to standard operative care (5420 mL vs. 4775 mL) [55].

**Fig. 1:**
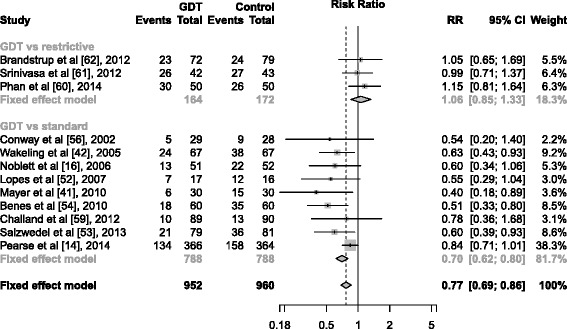
Meta-analysis of number of patients developing complications after abdominal surgery in studies using GDT. Some studies do not provide information on complications and are therefore excluded in the meta-analysis. Test for heterogeneity is significant, and the results should be interpreted with caution. Size of data marker corresponds to weighting of each study and RR with 95 % CI. *Diamonds* sum up the overall effect estimate. RR <1 favors GDT. Heterogeneity: tau^2^ = 0.04; chi^2^ = 20.41; *df* = 11 (*p* = 0.04); *I* ^2^ = 46 %. Test for overall effect: *z* = 4.56 (*p* < 0.0001)

However, not all trials showed a benefit [59–62]. In a study of 179 elective colorectal surgical patients subdivided into aerobically fit or unfit groups, Challand et al. demonstrated an impaired outcome with prolonged LOS and increased number of intensive care unit (ICU) admissions in the GDT group compared to standard care (5309 mL vs. 4010 mL) [59]. In a recent ambitious multicenter trial of 734 high-risk patients undergoing major gastrointestinal surgery, Pearse et al. randomized patients to a GDT algorithm using intravenous fluids and dopexamine vs. usual care (4190 mL vs. 4024 mL). They showed no significant improvement in the composite primary outcome consisting of 30-day mortality and complications [14]. However, an updated meta-analysis of randomized clinical trials testing GDT in abdominal surgery shows a significant reduction of patients developing complications when using a GDT approach (see Fig. 1).

It is important to note that diverse methodology, different patient categories, and the use of supplemental crystalloids in both the GDT and the reference group challenge comparison between studies and might explain the heterogeneity in results. Overall beneficial outcomes to GDT appear to be related to patient risk stratification, being more beneficial in groups with higher mortality rates and more comorbidities [63].

The GDT approach is usually applied in addition to “the standard fluid therapy”, compromising the ability of GDT to limit excessive fluid administration, allowing continuous intravenous crystalloid infusion alongside GDT optimization. Crystalloid infusion seems to have an insignificant effect on GDT measurements. This is in accordance with the findings by Lobo et al. and McIlroy and Kharasch, who showed a lower effect of crystalloids on the circulating volume and cardiac output. Hence, “the standard fluid therapy” or “maintenance regimen” should only replace physiological fluid turnover and pathological fluid losses with fluids resembling the loss in quantity as well as quality. The physiological loss is no more than 1–1.5 mL/kg/h substituting diuresis and insensible perspiration and is more than replaced by the fluid given with the different anesthetic and antibiotic medication.

Interestingly, recent studies comparing restrictive or zero-balance fluid therapy with GDT based on a zero-balance maintenance regime (categorized as “zero-balance GDT”) have shown no difference in outcome between the two approaches (see Table 2) [60–62, 64].

**Table 2:** Trials of “goal-directed fluid therapy” (GDT) in abdominal surgery versus “zero-balance fluid therapy” (restricted)

Author	Surgery	No. of patients/ASA	Blinding/monitor/timing	Primary outcome	Intervention	Preoperative fluid volume, mL (restricted vs. GDT)	Intraoperative fluid volume, mL (restricted vs. GDT)	Postoperative fluid volume, mL (restricted vs. GDT)	Effect of GDT
Brandstrup et al. [62]	Elective laparoscopic or open colectomy	150/ASA I-III	Observer blinded/ODM, CardioQ®/intraoperative	Postop. complications	Optimizing SV (<10 %) with HES 6 %, 130/0.4 (Voluven®)	2 h fasting for fluid 500 mL saline if no fluid in 6 h	Coll: 475 vs. 810 Cryst: 443 vs. 483 Total^a^: 1491 vs. 1876	Early oral intake in an enhanced recovery protocol. Iv-fluid if oliguria, tachycardia or hypotension	→ Complications → LOS Mortality: 1 (restricted) vs. 1 (GDT)
Zhang et al. [64]	Elective open GI surgery	60 in three groups: 4 mL/kg/h RL + GDT-HES, 4 mL/kg/h RL + GDT-RL, and 4 mL/kg/h RL alone/ASA I-II	Observer blinded/Datex Ohmeda®/intraoperative	LOS	Optimizing PPV (<11 %) with Ringer’s lactate and HES 6 %	Not given	Total volume: GDT-HES: 1742 vs. GDT-RL: 2109 vs. RL alone: 1260	1.5–2.0 mL/kg/h crystalloid for 3 days Oral intake not mentioned.	↓ LOS in GDT-HES ↓ Time to flatus in GDT-HES → Complications between groups Mortality: none
Srinivasa et al. [61]	Elective laparoscopic or open colectomy	85/ASA I-III	Observer blinded/ODM, CardioQ®/intraoperative	Surgical recovery score (SRS)	Optimizing cFT (<350 ms) and SV (<10 %) with Gelofusine	13 patients with bowel preparation: 1000 mL crystalloid	Coll: 297 vs. 591 Total^a^: 1614 vs. 1994	Early oral intake in an enhanced recovery protocol. IV fluid if oliguria, tachycardia, or hypotension	→ SRS → LOS → Complications Mortality: none
Phan et al. [60]	Elective colorectal surgery	100/ASA I-III	Observer blinded/ODM, CardioQ®/intraoperative	LOS	Optimizing cFT (<360 ms) and SV (<10 %) with any colloid	400 mL PreOp® the day before and 2 h preop.	Coll: 0 vs. 500 Cryst: 1400 vs. 1500 Total^a^: 1500 vs. 2190	Early oral intake in an enhanced recovery protocol	→ LOS → Complications Mortality: 1 (restricted) vs. 0 (GDT)

*LOS* length of hospital stay, *ODM* Oesophageal Doppler Monitoring, *SV* Stroke Volume, *PPV* Pulse Pressure Variation, *cFT* corrected Flow Time

^a^Total volume infused including colloid, crystalloid and blood products

↑significantly increased, ↓ significantly decreased, → no significant changes

Brandstrup et al. randomized 151 patients to zero-balance GDT compared to a zero-balance fluid approach (1876 mL vs. 1491 mL) and showed no difference in mortality and postoperative complications, despite a significant increase in SV in the GDT group. Likewise, Srinivasa et al. randomized 85 patients undergoing colectomy to GDT vs. a restrictive regime (1994 mL vs. 1614 mL) and found superior cardiac indices in the GDT group, but no difference in surgical recovery, LOS, and complications per patient. In the same way, Phan et al. showed improved stroke volume index but no difference in LOS in a study of 100 patients randomized to GDT vs. restrictive therapy (1500 mL vs. 1400 mL). Thus, zero-balance or restrictive fluid approach seems equal compared to the zero-balance GDT approach during elective abdominal surgery with a RR, 1.06 (95 % CI, 0.85–1.33) (see Fig. 1).

### Trials of outpatient surgery

The trials of outpatient abdominal surgery are shown in Table 3 [65–71].

**Table 3:** Trials of outpatient abdominal surgery

Author	Surgery	No. of patients	Blinding	Duration of surgery	Intervention	Fast	Postop. oral fluid intake	Effect of fluid
Keane and Murray [65]	Mixed outpatient surgery	212 in 2 groups	No	18 min	1000 mL Hartman’s solution + 1000 mL DW vs. no fluid	?	?	↓ Thirst, drowsiness, headache and dizziness → Nausea
Spencer [66]	Minor gynecologic surgery	100 in 2 groups	No	8 min	1 L CSL vs. no fluid	?	?	↓ Dizziness and nausea
Cook et al. [67]	Gynecologic laparoscopy	75 in 3 groups	Yes	20 min	CSL 20 mL/kg vs. CSL + DW 20 mL/kg vs. no fluid	11–16 h	?	↓ Dizziness and drowsiness ↓ LOS in Dextrose group
Yogendran et al. [70]	Mixed outpatient surgery	200 in 2 groups	Yes	28 min	Plasmolyte 20 mL/kg (1215 mL) vs. Plasmolyte 2 mL/kg (164 mL)	8–13 h	?	↓ Thirst, dizziness and drowsiness → PONV
McCaul et al. [71]	Gynecologic laparoscopy	108 in 3 groups	Yes	22 min	CSL 1,5 mL/kg/fasting h (1115 mL) vs. CSL + DW 1.5 mL/kg/fasting h (1148 mL) vs. no fluid	11,5 h	?	→ PONV ↑ Thirst in CSL + DW group
Magner et al. [68]	Gynecologic laparoscopy	141 in 2 groups	Yes	20 min	CSL 30 mL/kg vs. CSL 10 mL/kg	13 h	?	↓ PONV → Dizziness and thirst
Holte et al. [69]	Laparoscopic cholecystectomy	48 in 2 groups	Yes	68 min	LR 15 mL/kg (998 mL) vs. 40 mL/kg (2928 mL)	2 h	Mean 600 mL	↓ LOS ↓ Thirst, nausea, dizziness, and drowsiness ↑ Well-being and pulmonary function

*DW* Dextrose in water 5 %, *CSL* compound sodium lactose (Na:131, K:5, Ca:2, Cl:111, Lactate:29 mmol/l), *LR* lactated Ringers solution, *PONV* postoperative nausea and vomiting

↑significantly increased, ↓ significantly decreased, → no significant changes, ?: not given

In 1986, Keane and Murray investigated fluid therapy in outpatient surgery and showed reduced thirst, drowsiness, headache, and dizziness in the group receiving 1 L of Hartmann’s solution and 1 L 5 % dextrose preoperative compared to patients without fluids [65]. In comparison, McCaul et al. demonstrated no difference in postoperative nausea and vomiting (PONV) between 108 patients undergoing gynecologic laparoscopy randomized into three groups receiving no fluid, 1.5 mL/kg/fasting hour of compound sodium lactate (CSL) or 1.5 mL/kg/fasting hour CSL with an additional 0.5 g/kg of dextrose [71]. In contrast to this finding, Magner et al. randomized 141 patients undergoing gynecologic laparoscopy and found reduced nausea and vomiting in the group receiving 30 mL/kg of CSL compared to 10 mL/kg CSL [68]. Despite the discrepancy, a tendency towards reduced PONV, dizziness, and drowsiness seems related to intravenous infusion of 1–2 L of crystalloids in outpatient surgery, an amount comparable to the fasting deficit.

One trial by Holte et al. stands out, being the only one showing a beneficial outcome in the group receiving 2928 mL compared to 998 mL (40 mL/kg vs. 15 mL/kg) for patients undergoing laparoscopic cholecystectomy. Patients showed an improvement in postoperative nausea and vomiting, performance on a treadmill, and balance test in the group receiving the most fluid [69]. However, a significantly increased administration of postoperative opioids in the restricted group most likely affected the outcome parameters.

## Conclusions

Oral fluid intake should be encouraged up to 2 h prior to surgery, thereby minimizing the need for intravenous compensation. Preferably, carbohydrate-containing fluids should be given due to patients’ proven reduction of postoperative insulin resistance and improved well-being.

Perioperative fluid turnover accounts for no more than 1–1.5 mL/kg/h consisting of diuresis, insensible perspiration, evaporation from the wound, and accumulation in the traumatized tissue and should be compensated by carbohydrate-containing (hypotonic) fluids unless counter indications are present. Sensible perspiration varies considerably and is recommended replaced by balanced crystalloids. The assumption that elective surgery causes a fluid loss to the third space is based on flawed methodology and the replacement of a “loss to third space” worsens the postoperative outcome, due to derived fluid overload. Hence, this practice should be abandoned. A delicately balanced fluid therapy is recommended to avoid adverse effects of unnecessary excessive fluid administration as edema, inflammation, and compromised tissue healing.

The intraoperative zero-balance fluid approach based on measurement of lost blood and fluid and postoperatively on body weight is easily implemented and has been shown to reduce postoperative major and minor complications. Therefore, a zero-balance fluid approach is recommended in the elective perioperative setting. A GDT approach likewise has shown to improve postoperative outcome, and guidelines recommending GDT seem well supported. However, the GDT practice is not documented to be superior to the zero-balance fluid approach. Nevertheless, high-risk surgery with multimorbid patients might benefit from the dynamic GDT approach. Evidence regarding urgent surgery is lacking, leaving a gap for future studies to explore.

In relation to outpatient surgery, 1–2 L balanced crystalloids reduces PONV and improves well-being.

## Abbreviations

CSL: compound sodium lactate
GDT: goal-directed fluid therapy
HES: hydroxyethyl starch 6 %
ICU: intensive care unit
LOS: length of hospital stay
PONV: postoperative nausea and vomiting
